# The Period between School-Leaving and Hospital Training

**Published:** 1921-07-16

**Authors:** F. A. Sheldon

**Affiliations:** Guy's Hospital Trained Nurses' Institution, S.E. 1


### The Period Between School-Leaving and Hospital Training.

*To the Editor of* The Hospital.

Sir,—At the present time the draft Syllabus of lectures and demonstrations for education and training in general nursing is receiving the earnest attention of matrons, medical men, and hospital boards throughout the country. This most admirable Syllabus is, however, entirely designed for use in hospitals and for the woman of twenty-one years of age (or at the youngest nineteen) who has definitely decided upon a nursing career. Would the General Nursing Council consider the desirability and possibility of arranging a special course, based upon the Syllabus, which the girl of eighteen could take on leaving school at a university, college, or polytechnic? Entrance examinations and scholarships for this course would attract the type of girl we want, and would obtain the interest of her teachers. In most cases she could live at home, not losing too soon its influence and pleasures, and this would help to win the approval of her parents.

The nursing diploma obtained (by examination) should be recognised by the General Nursing Council, and at nineteen the student would enter hospital with this knowledge accredited to her. The time thus saved could be used towards the end of her training to specialise in, say, public health or administrative work, etc. This University course would also obviate the establishment of so many preliminary hospital schools.

We cannot hope to improve our present position without an adequate (contributory) superannuation scheme entailing a longer wage-earning life, and this may be accomplished by linking up the actual entrance into hospital with school-life.—Yours faithfully, F. A. Sheldon.

Guy's Hospital Trained Nurses' Institution, S.E. 1,
July 11, 1921.

